# Sociotechnical Drivers and Barriers in the Consumer Adoption of Personal Health Records: Empirical Investigation

**DOI:** 10.2196/30322

**Published:** 2021-09-24

**Authors:** Umar Ruhi, Armin Majedi, Ritesh Chugh

**Affiliations:** 1 Business Analytics & Information Systems Telfer School of Management University of Ottawa Ottawa, ON Canada; 2 University of Ottawa Ottawa, ON Canada; 3 College of Information & Communication Technology School of Engineering & Technology Central Queensland University Melbourne Australia

**Keywords:** electronic personal health records, PHR, patient facing information systems, technology adoption, technology acceptance, consumer health informatics

## Abstract

**Background:**

Increasingly popular in the health care domain, electronic personal health records (PHRs) have the potential to foster engagement toward improving health outcomes, achieving efficiencies in care, and reducing costs. Despite the touted benefits of PHRs, their uptake is lackluster, with low adoption rates.

**Objective:**

This paper reports findings from an empirical investigation of the sociotechnical factors affecting the adoption of PHRs.

**Methods:**

A research model comprising personal and technological determinants of PHR adoption was developed and validated in this study. Demographic, technographic, and psychographic data pertaining to the use of PHRs were collected through a web-based questionnaire for past, current, and potential users. Partial least squares-based structural equation modeling was used to estimate a structural model of cognitive and affective factors impacting intentions to use PHRs.

**Results:**

The analysis revealed that in addition to the expected positive impact of a PHR system’s usefulness and usability, system integration also positively affects consumers’ intention to adopt. The results also suggest that higher levels of perceived usability and integration do not translate into higher levels of perceived usefulness. The study also highlights the importance of subjective norms, technology awareness, and technology anxiety as direct antecedents of the intention to adopt PHRs. The differential effects of the adoption factors are also discussed.

**Conclusions:**

We hope that our study will contribute to the understanding of consumer adoption of PHRs and help improve the design and delivery of consumer-centric health care technologies. After discussing the implications for research, we provide suggestions and guidelines for PHR technology developers and constituents in the health care delivery chain.

## Introduction

### Background

Within the realm of health systems and applications, electronic personal health records (PHRs) represent a burgeoning technology that is gaining traction in many countries worldwide [1-5]. As a consumer-centric technology, a PHR can be defined as “an electronic application through which individuals can access, manage, and share their health information and that of others for whom they are authorized, in a private, secure, and confidential environment” [6]. In this regard, PHR systems comprise information and communication technologies that can potentially help all types of end users maintain health and wellness [7], and specifically facilitate patients to manage their ongoing illnesses [8].

In this paper, we characterize PHR technologies as those specifically pertaining to digitally stored health care information about an individual patient under the control of that patient or their caregiver [5,9]. This is in contrast to other technologies, such as electronic medical records (EMRs) and electronic health records (EHRs) that are typically maintained by health care providers or payor organizations [10]. Furthermore, our discussion applies to various forms of PHR systems identified in the extant literature, including stand-alone PHRs that require users to manually enter their health data and medical history [8,11,12], tethered PHRs that are offered as an extension of a health institution’s back-end EHR or EMR [8,11,13], and interconnected PHRs that offer interoperability across various health information systems (HISs) [11,14].

Industry analysts have predicted great market potential for PHR-related technologies. For instance, according to studies conducted by the Markle Foundation, over 70% of US health care consumers believe that PHRs can improve health care quality [6,15]. Similarly, a study by Deloitte [16] highlighted that more than half of the US adult population may be interested in using web-based PHR services.

At the macro level, leveraging the potential value of PHRs in facilitating patient engagement and improving consumer health outcomes has been a key constituent of several government eHealth initiatives around the world. For example, in the United States, the Health Information Technology for Economic and Clinical Health Act established a meaningful use incentive program offering financial support to providers and health systems adopting EHR-related technologies [17]. Meaningful use stage 2 specifically calls for technologies that facilitate patient engagement in terms of personal health information management and care coordination, whereas stage 3 extends the requirements for these systems to include patient communication functions, patient education features, and interoperability with back-end EHRs [17,18]. Similarly, the European Union has funded several eHealth infrastructure projects with the aim of supporting personalized medicine, including the p-medicine EU project and the eHealthMonitor project [5]. Along similar lines in Canada, the Canada Health Infoway sponsors several federally funded projects to promote the adoption of consumer-focused digital health technologies ranging from health information records to patient-physician communication and remote patient monitoring [19].

Notwithstanding the industry forecasts about abundant consumer interest and government commitments to PHR technologies, the adoption of these technologies has been much slower than originally expected [4,20]. This disconnect between active interest and low actual use has been termed the *PHR paradox* [21]. Various reasons for lackluster adoption have been cited in the extant literature, often contradicting intuition, and sometimes with inconsistent findings across studies [22-25]. Consequently, many researchers have called for further studies in the area of consumer adoption of PHRs [21,22,25-27]. Our research aims to answer this call and further explore and clarify the role of sociotechnical factors in the adoption of PHRs.

In delineating the scope of investigation of this study, we would like to highlight our deliberate use of the term consumer instead of patient throughout the discussion. Our objective is to investigate factors that impact the adoption of PHRs from the perspective of all users who may be current as well as potential users of these systems. Toward this, we aim to include not only users who are currently receiving active care (patients), but also those who may simply be interested in maintaining their health information and medical history, or in using other nonclinical functionalities of PHRs (consumers). Other academic researchers and industry analysts have also commented on the distinction between patients and consumers, noting that consumers may include both current and prospective patients [28]. Moreover, consumers often have more decision-making flexibility than patients because the latter are primarily concerned with the management of their specific medical conditions [29-31].

By virtue of its orientation, this research study is principally situated in the field of consumer health informatics (CHI), a field concerned with health and health care-related preferences and information needs of consumers and associated medical and public health practitioners [32,33]. Technology applications such as PHRs, which can help empower consumers to manage their own health, constitute an important focus of attention in the CHI field [14,26,34]. In this study, we seek to explore various personal and technological factors that can affect the adoption of PHR tools and applications, identifying with the broad objectives for CHI research toward analyzing, modeling, and integrating consumer preferences into medical information systems (ISs) [35].

### Related Work

Researchers who have investigated user adoption of PHRs have suggested that possible adoption barriers may be related to technology factors, such as privacy and security concerns, system usability, and poor integration with health care provider systems [36,37]. Furthermore, personal factors, such as inadequate technology competency, low technology awareness, unrealistic expectations, and presence of chronic medical conditions, have also been linked to the likelihood of adoption of these technologies [38-40]. Some of these factors have been empirically validated, but the results across investigations are often inconsistent [23-26,41-43].

Consequently, researchers have called for further empirical studies to explore and validate the role of specific PHR adoption factors. Multimedia Appendix 1 [8,22,24-26,39,41,44-57] provides a chronological summary of research studies in the area of PHR adoption and outlines key takeaways from each study. Specific calls for further research in each study are also highlighted.

Our review of the extant literature indicates that patients with chronic illnesses or disabilities, their caregivers, and people caring for older persons are more likely to adopt and use PHR technologies [15,44-46,58-61]. These groups of users will find PHR technologies useful as a communication tool to obtain personalized care from their clinicians [7,47-50,59] and as an organizational tool to help track patient health conditions, maintain medication lists, write patient diaries, and keep notes from physician consultations [7,8,41,49,50,60,62].

Current research also shows that factors such as computer anxiety, security and privacy concerns, and perceptions of usefulness are key determinants of PHR adoption across different consumer strata [22-24,43,51-54,63]. In contrast, research on several adoption factors, such as usability perceptions, consumer health literacy, and user self-efficacy, has shown varied and inconsistent results in the extant literature. For example, in multiple studies, Archer and Cocosila found different results pertaining to the impact of health-information seeking preferences and self-efficacy of individuals on the adoption of PHR systems [22,23,51].

In terms of key areas for further exploration, our review indicates the need for more research on PHR adoption along several lines. From the perspective of personal factors, there is a significant lack of empirical evidence on the role of social influence processes in PHR adoption. In our review, we found only two studies that investigated the role of subjective norms in the adoption of PHRs [52,64]. On the technology side, very few studies have empirically validated the role of usability perceptions and system integration attributes as part of the cognitive instrumental processes that impact PHR adoption. With respect to the former, only a few studies have investigated usability through the limited lens of perceived ease of use [24,52,55,56] despite anecdotal evidence and expert opinion that suggests that PHR usability includes additional dimensions [25,26,65]. Our study aims to address these gaps in the extant literature by conceptualizing these key factors and their relationships with other PHR adoption determinants. The next section describes our research model and its underlying constructs and hypotheses.

### Research Model and Theoretical Underpinnings

#### Overview

Notwithstanding the differences in results across some studies, researchers continue to investigate factors impacting consumer adoption of PHRs with the aim of improving our cumulative understanding of this phenomenon. As such, additional research in this area has been recommended by many researchers to further explore the impact of personal, technological, organizational, and environmental factors on consumer acceptance of PHR technologies, including patients and their caregivers [21,24,25,48,66].

**Figure 1:**
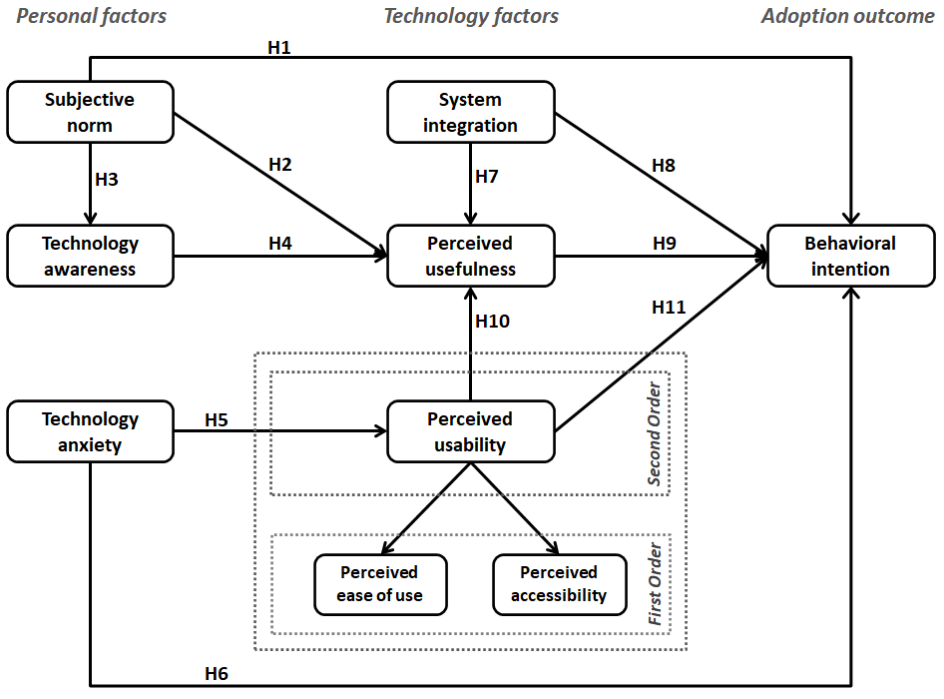
Research model and construct definitions.

This paper answers the call by theorizing and validating the role of various personal and technological factors as possible determinants of PHR adoption. We aim to contribute to the body of knowledge on the adoption of PHR systems by exploring sociotechnical factors that not only further clarify or complement those previously studied by other researchers, but also offer new avenues of inquiry. The scope of our investigation includes the study of subjective norms, technology awareness, and technology anxiety as personal factors affecting PHR adoption, and system integration, perceived usefulness, and perceived usability as technological antecedents of PHR adoption. These constructs and their definitions are provided in Table 1, and their posited interrelationships are shown in Figure 1. The theoretical justification for all research model constructs and hypotheses is outlined in the following subsections.

**Table 1:** Research model constructs.

Theme and constructs		Conceptual definition
**Personal factors (determinants)**		
	Subjective norm	The degree to which users perceive that most people who are important to them think they should or should not use the system [67,68]
	Technology awareness	An individual’s familiarity with the purpose and benefits of the technology [69,70]
	Technology anxiety	An individual’s apprehension or fear when confronted with the use of technology [71,72]
**Technology factors (determinants)**		
	System integration	Extent of connection and interoperability among technology components and subsystems [73]
	Perceived usefulness	The degree to which users believes that using the system will help them toward achieving their desired goals [68,74]
	Perceived usability (ease of use and accessibility)	The degree of ease associated with the system [68,74] Intuitive interface and information structure that is comprehensible and available when needed [75,76]
**Adoption** **outcome (consequent)**		
	Behavioral intention	The degree to which a person has formulated conscious plans to perform or not perform some specified future behavior [68]

#### Subjective Norm

In technology adoption studies, the concept of subjective norm is appropriated to account for social influences that impact a potential user’s decision to adopt and use a technology. The concept of subjective norm has its theoretical underpinnings in the theory of reasoned action, which defines it as “person’s perception that most people who are important to him think he should or should not perform the behavior in question” [67]. In technology adoption studies, subjective norm represents perceived social pressure to use a new technology [68,71] and has been shown to be a significant determinant of behavioral intention to use a technology [77,78].

In the context of PHR system adoption, there is a dearth of research exploring the role of social influence on a user’s decision to adopt these technologies. In our literature review, we identified one study that investigated subjective norms in the context of hardware-based (USB) PHRs within the specific regional context of Taiwan [52], and one study in Thailand, in which social influence was key in influencing the use of PHR [64]. As such, we expect subjective norms to play an even greater role as an antecedent of adoption for web-based PHRs, given that web-based technologies are likely to diffuse faster than hardware technologies. In addition, in this study, we aim to investigate whether subjective norm has only a direct impact on use intention, or whether it also plays an important role in internalizing the benefits of PHR technologies by affecting individual perceptions of the usefulness of these technologies. The following hypotheses related to subjective norms are posited in our research model:

- H1: Favorable subjective norms pertaining to the use of PHR technologies have a positive effect on the behavioral intention to use PHRs.
- H2: Favorable subjective norms pertaining to the use of PHR technologies have a positive effect on the perceived usefulness of PHRs.

#### Technology Awareness

Despite PHR technologies having been introduced more than a decade ago, research has found that there is a lack of awareness about them among many potential end users [49,54,56,79-82], thus inhibiting their use. This lack of awareness about PHR technologies has also been attributed to people having unrealistic expectations of these technologies [26,39,83], leading to their abandonment. A report by the Office of the National Coordinator for Health Information Technology [80] also found that people in the United States were especially unaware of stand-alone PHR offerings because they do not get similar promotional exposure as to health care institution-sponsored tethered PHR systems. Given the repercussions the lack of awareness can have on PHR adoption, several researchers have stated the need to address this research gap [26,79,84], and further posit calls for further research into the promotion of PHRs [82,85], including strategic wording [86] and educational or training programs [54,81]. Toward this, we draw upon the consideration of adoption studies conducted in the realm of other technologies to explore the role of technology awareness as a prerequisite to the development of perceptions about PHR usefulness [69,78].

In addition to exploring the role of technology awareness as a direct antecedent of perceived usefulness, we also explored its relationship with subjective norms. Research literature on the diffusion of innovation considers interpersonal relationships as an effective channel for creating awareness about an innovation [87,88]. These interpersonal channels can help create awareness by emphasizing the personal value of an innovation to a potential adopter [69]. We expect this to be the case for PHR technologies. The following two hypotheses related to technology awareness were tested in our research model:

- H3: Favorable subjective norms pertaining to the use of PHR technologies have a positive effect on technology awareness of PHRs.
- H4: Greater technology awareness of PHR technologies has a positive effect on the perceived usefulness of PHRs.

#### Technology Anxiety

Previous research in ISs shows technology anxiety to be a significant barrier to the adoption of new technologies [71,89], and the same findings have been echoed in research on the adoption of PHR systems [22,51]. However, a majority of PHR research to date simply considers the direct impact of technology anxiety on a user’s intention to adopt PHRs without exploring its indirect effect on adoption through other key antecedents such as perceived ease of use. Past IS research shows technology anxiety to be an emotional anchor that leads to negative expectations of a technology [90], especially during the initial stages of its adoption. Previous IS studies have validated the importance of anxiety as an antecedent of perceived ease of use [91,92].

To address this gap in PHR adoption research, our model posits technology anxiety as an affective construct that affects the adoption of PHRs. We explored the direct link between anxiety and behavioral intention and its indirect effect on perceptions of usability (ease of use and accessibility). In doing so, our model attempts to capture the varying causes and effects of anxiety expressed in the extant literature on PHR adoption. These include inadequate technology literacy [27,59,81], individual uneasiness with setup of in-person authentication for tethered PHRs, lack of technical ability to integrate multiple data sources into stand-alone PHRs [80], or a general fear of technology [48,53]. In summary, we propose that technology anxiety potentially plays an important role in shaping cognitive responses toward PHR systems and directly affects behavioral intention to use these technologies. The following two hypotheses related to technology anxiety are proposed:

- H5: A higher level of technology anxiety has a negative effect on the perceived usability of PHRs.
- H6: A higher level of technology anxiety has a negative effect on the behavioral intention to use PHRs.

#### System Integration

Among the various contemporary PHR architectures, one may expect greater consumer interest in interconnected PHRs rather than stand-alone PHRs or even tethered PHRs. It is our position that with greater access to health and medical information available through multiple sources, consumers may be more motivated to use PHR systems. Such systems are likely to garner more interest through their *one-stop shopping* appeal, offering users a unified view of their health and medical information across the health care delivery chain.

Although many researchers and industry experts have commented on the lack of interoperability as a major barrier to consumer adoption [11,12,93-96], our literature review did not reveal any empirical substantiation of this conjecture. To address this issue, our research model incorporates system integration as a posited antecedent of perceived usefulness, as well as a direct determinant of behavioral intention. By exploring these relationships, we aim to investigate whether system integration aspects of PHRs are internalized through gradual system use, hence shaping user perceptions of usefulness, or whether the system integration factor is more prominent as an upfront reason to adopt or reject a PHR system. We offer the following two hypotheses related to system integration:

- H7: Greater system integration in PHR technologies has a positive impact on the perceived usefulness of PHRs.
- H8: Greater system integration in PHR technologies has a positive impact on the behavioral intention to use PHRs.

#### Perceived Usefulness

The extensive body of knowledge on the technology acceptance model (TAM) [74,97] shows that perceived usefulness is one of the strongest determinants of technology adoption [68,71,78]. Therefore, we expect perceived usefulness to be a strong determinant of PHR system adoption. Previous research on PHR adoption has validated the important role of perceived usefulness as a predictor of adoption [23,45,51,84,98]. In our model, we use perceived usefulness to signify performance expectancy in the use of PHRs, that is, the belief that using PHR will help in managing personal health. Furthermore, we also appropriately perceived usefulness as a cognitive response construct that is affected by other personal and technological determinants of PHR adoption. In addition to the previously posited hypotheses with perceived usefulness as the consequence (H2, H4, H7), we retained the conventional TAM hypothesis:

- H9: The higher perceived usefulness of PHR technologies has a positive impact on the behavioral intention to use PHRs.

#### Perceived Usability

Our final technological construct in the research model is theorized as a multidimensional factor consisting of the dimensions of perceived ease of use and perceived accessibility. The perceived usability construct in our model aims to capture the notion of effort expectancy associated with PHR systems, that is, the degree of ease associated with using PHRs.

The traditional view of the perceived ease of use construct in TAM also signifies effort expectancy [68,71]. However, research has shown that effort expectancy is usually a combination of ease of use and other contextual factors that shape end user perceptions about the relative difficulty of understanding and using the system [71]. In the context of PHR technologies, we believe that accessibility is a contextual factor that impinges effort expectancy. Research on PHR adoption factors indicates that aspects related to the intuitiveness of the user interface, understandability of information, availability through multiple channels (eg, desktop, web and mobile), and convenience of anytime anywhere access are important factors that affect individual perceptions of usability of PHR systems [8,65,99,100]. As such, our conceptualization of perceived accessibility attempts to assess the significance of these elements in determining end user perceptions of the usability of PHR technologies. To our knowledge, no previous research on PHR adoption has corroborated the role of accessibility in the acceptance of these technologies.

In conceptualizing perceived usability, we retain *ease of use* as an underlying dimension because it relates directly to other aspects of software usability, including end user efficiency and learnability with the system [101,102]. Furthermore, although previous research on PHRs has commented on the importance of ease of use for PHR adoption [53,56,83,95,96,103,104], very few studies have explored its role in the nomological network of other cognitive, affective, and behavioral factors [52,105]. On the basis of our multidimensional conceptualization of perceived usability, we propose the following two hypotheses:

- H10: Greater perceived usability of PHR technologies has a positive impact on the perceived usefulness of PHRs.
- H11: Greater perceived usability of PHR technologies has a positive impact on the behavioral intention to use PHRs.

#### Behavioral Intention

To characterize the adoption of PHRs, we used behavioral intention as the ultimate downstream construct in our research model. As a critical outcome of various cognitive and affective antecedents, this construct has its original basis within the theory of reasoned action [67], which conceptualizes it as a consequence of individual beliefs and as an antecedent of actual behavior. The construct has been commonly deployed in the IS literature to study the adoption of various types of technologies [74,97] including PHRs [22,23]. Furthermore, within the context of health behaviors, past research indicates that behavioral intention is significantly correlated with actual use [106-108]. Therefore, we expect greater behavioral intention to correspond to higher levels of actual use of PHR systems.

Overall, our research model aims to offer an inclusive basis for validating the role of three different types of determinants on PHR adoption—(1) individual differences, (2) system characteristics, and (3) social influence. Research models that include these categories of factors have been recommended as a practical foundation for investigating the adoption of new technologies [109]. It should be noted here that although we intend to be inclusive of these categories, we do not claim to be exhaustive over all possible adoption factors. As such, other adoption factors such as security and privacy concerns and health literacy have already been investigated in previous research studies, with largely consistent findings about the importance of these factors [22,51,84,110].

In terms of organization, our empirical methodology is described in terms of key procedures, and the results of our investigation are outlined. Finally, the discussion and conclusion sections offer an interpretation of the results, especially with respect to their implications for research and practice.

## Methods

### Survey Questionnaire Content

The research model posited in the previous section was validated through a quantitative empirical investigation using a web-based survey instrument. Details of the survey content, measurement scales, analysis procedures, and data collection techniques are presented below.

The survey comprised *demographic* information questions about the respondents’ age, gender, and country of residence; *technographic* behavioral items related to respondents’ experience and interest in using PHR technologies, as well as their preferences for different PHR features and functions, and *psychographic* questions pertaining to different constructs in the research model. For the latter, each construct in the research model was operationalized using multi-item psychometric scales with Likert-scale questions. Where possible, the items for a construct were adapted from previously validated measurement scales. We created new items for *system integration* and *perceived accessibility* constructs and modified the wording of items related to other constructs to align with the context of PHR systems.

To develop the two new scales, various qualitative and quantitative content validity assessment procedures were used, including concept elicitation interviews with subject matter experts (n=7) to generate representative and relevant measurement items; cognitive interviews with potential respondents from the target sampling frame (n=5) to ensure item relevance and clarity, and the final selection of measurement indicators based on item relevance ratings of subject matter experts, which were subsequently used to calculate item-level content validity indices (I-CVI). Drawing upon recommendations from the extant literature [111-113], a conservative cutoff value of 0.80 was used for item-level content validity indices to select items for the new scales. The 7 people in the subject matter expert panel included 2 faculty members from the health informatics domain at the authors’ home institution, 1 health information technology business analyst working in a government agency, 2 doctoral students specializing in health information technology interoperability, one experienced end user of a PHR system, and a website manager of a patient portal of a health care institution.

At the end of the survey, participants were also invited to optionally respond to this open-ended question about PHR use: “Do you have any other comments about the use of personal health records (PHRs)? What factors do you consider to be important in your decision to start using or keep using technologies such as PHRs?”

The complete survey instrument was assessed for face validity through consultations with other HIS researchers, and construct validity for each theoretical construct was assessed through exploratory factor analysis of the pilot survey responses (n=20). Multimedia Appendix 2 [70,71] lists the final survey measurement items used for each construct in the research model.

### Data Collection

Data for this study were collected through a web-based survey administered to actual and potential users of PHR technologies. Screening questions were asked at the beginning of the survey to determine different classes of respondents, and a brief overview of PHR technologies was offered to ensure qualified responses. As outlined in Multimedia Appendix 2, two alternative versions of questions were used to elicit responses from potential and actual (past or current) users of PHR systems.

The sampling techniques used were primarily based on convenience and self-selection. We recruited respondents who had basic familiarity with PHRs or similar tools for health care self-management. We used a two-pronged approach for data collection to ensure a cross section of potential PHR consumers. First, we solicited participation from current and past users of a PHR portal sponsored and supported by a teaching hospital (tethered PHR) in Ontario, Canada. In distributing our call for participation, we emphasized our interest in obtaining responses from current and past users of the PHR system. Second, calls for participation were also communicated through various web-based forums and social media groups dedicated to the discussion of health-related topics. To ensure a diverse selection of respondents, our sampling frame included both general health and wellness sites, as well as sites for chronic illness support groups. Once again, we underlined our goal of including responses from existing and potential users of PHR technologies.

Permission was sought from site administrators or forum moderators before posting our call for participation. In the case of the hospital PHR, our call for participation was distributed by the administrator to a mailing list of PHR users who had opted to receive news and information from the website at the time of their registration with the portal. No respondent incentives were offered for completing the survey.

The survey responses were collected over a 4-week period, with one reminder posted at each site with the original call for participation. Key suggestions from the Dillman tailored design method [114] were used to promote response rates for the survey. These included customizing the call for participation according to each site and posting personalized answers to any questions posted by potential respondents in a timely fashion. An interactive approach to collecting web-based survey data has been suggested by various researchers [115,116].

Because partial least squares (PLS) was the planned multivariate statistical analysis procedure in this study, the minimum sample size heuristic for PLS studies [117,118] was used for an a priori calculation of the required sample size. Using this heuristic, the minimum target sample size for this study was determined to be 60 valid responses. The heuristic suggests that the minimum sample size requirement for PLS- based models is determined by finding the larger of the following values: (1) 10 times the largest number of antecedent variables that affect any consequent in the model, or (2) 10 times the number of maximum indicators (manifest variables) in a latent variable in the model [117,118]. For the theoretical model under investigation, the *Behavioral Intention* construct has 5 direct antecedents, whereas *Perceived Usability* has the most indicators assigned to its measurement, specifically 6 items as shown in Multimedia Appendix 2. Therefore, the minimum target sample size for this study was determined to be 60 valid responses.

### Analysis Procedures

Responses to demographic and technographic questions were analyzed using descriptive statistics and nonparametric statistical tests, and testing of research model constructs and hypotheses was conducted through exploratory factor analysis and PLS-based structural equation modeling (SEM) techniques. The PLS approach for SEM was selected for this study because of its suitability for small-sample exploratory research [119] and its flexibility with multivariate normality assumptions [120].

Testing for common method bias was achieved by using three different procedures—(1) the Harman post hoc one-factor test [121], (2) verification of latent variable correlations as recommended by Pavlou et al [122], and (3) the PLS-based common latent factor test suggested by Liang et al [123].

## Results

### Overview

A total of 224 responses were collected from various sources, including the hospital PHR portal, web-based forums, and social media groups in our sampling frame. After discarding partial responses, 168 responses were retained for further statistical analysis. This exceeded our minimum sample size target, as specified above. The results from our analysis of the survey responses are detailed in the following subsections.

### Demographic and Technographic Highlights

Table 2 provides a summary of the basic demographic and technographic information from the survey responses analyzed. A significant proportion of respondents indicated familiarity with PHR technologies, with many respondents indicating current or past use of PHRs. Overall, 62% of the respondents self-identified themselves as either patients or caregivers.

**Table 2:** Key highlights from the respondent sample (n=168).

Demographic and technographic factors			Frequency, n (%)
**Gender**			
	Female	96 (57.1)	
	Male	72 (42.9)	
**Age (years)**			
	18-25	22 (13.1)	
	26-35	31 (18.5)	
	36-45	66 (39.3)	
	46-55	28 (16.7)	
	55 or older	21 (12.5)	
**Respondents source**			
	PHR^a^ portal	59 (35.1)	
	Online health communities	109 (64.9)	
**PHR familiarity and use**			
	Familiar	116 (69.1)	
	Current use	64 (38.1)	
	Past use	30 (17.9)	
**Health status identification**			
	Patients	66 (39.3)	
	Caregivers	39 (23.2)	

^a^PHR: personal health record.

On the survey question pertaining to the importance of various health care issues, respondents consistently identified better clinical health care outcomes as the top priority for them. These were followed by issues surrounding better delivery of health care, including access and cost of health care, as well as better communication with physicians. Multimedia Appendix 3 shows the top 5 issues identified in our survey based on the mean importance of each health care issue. In addition, the figure shows the top 10 PHR features identified in our survey. On the basis of the mean utility scores ranging from 1 to 7, we can see that content-based features that allow consumers to exercise control over their medical information take precedence for most people, followed by connectivity features that facilitate patient-provider and patient-physician communication. Juxtaposed alongside each other, the health care issues that are top priority seem to be drivers for the use of many PHR features, for example, system features related to the management of chronic illnesses through tracking of health information and medical history were deemed extremely important overall.

The next section outlines the results of the assessment of psychographic variables in the posited research model. Following the two-step approach for SEM analysis suggested by Anderson et al [124], an examination of the measurement model was conducted before testing the structural model. Both the measurement and structural models were estimated using the SEM facilities of Smart PLS [125].

### Measurement Model Assessment

The measurement model was assessed through a combination of exploratory factor analysis procedures and various tests for discriminant and convergent validities for the constructs in the research model.

We assessed our multidimensional operationalization of the *perceived usability* construct through exploratory factor analysis. Using principal axis factoring with promax rotation, a two-factor model emerged with 3 out of 7 items loading on the first factor and 3 on the second factor, all above the threshold of 0.70. One item that did not load well on either factor was dropped, and the scale was recalibrated with the remaining items, three corresponding to *perceived ease of use*, and three loadings on *perceived accessibility*. Subsequently, *perceived usability* was operationalized as a reflective higher-order factor structure in our model. To this end, we applied the repeated indicators (superblock) technique [126], which is the most commonly used approach for estimating hierarchical component models in PLS [127].

For our main measurement model, we inspected the loading and cross-loading of the indicators, as presented in Multimedia Appendix 4, Table S1. The highest loading for each measurement item (shown in bold) corresponds to its respective latent variable, and these loading values were higher in comparison to the item cross-loading on other model constructs. Moreover, except in one case, the substantive loading of each item on its construct exceeded the recommended threshold of 0.70, indicating item reliability [118]. In the case of item T_Anx_3, where the loading was slightly below the threshold, because the loading was rounded up to 0.70, the item was retained to ensure content validity. Overall, the assessment of loading and cross-loading demonstrated satisfactory reliability and discriminant validity at the item level.

We also followed the Fornell and Larcker guidelines [128] to ensure that the theoretical model constructs were all distinct. A visual inspection of Multimedia Appendix 4, Table S2 shows that for each construct, the square root of the average variance extracted (AVE; shown in bold on the diagonal) exceeds other interconstruct correlations. This demonstrates the discriminant validity of our measurement model at the construct level.

Various tests of convergent validity were performed through an assessment of quality indices, as shown in Multimedia Appendix 4, Table S3. As shown, the AVE value for each construct is higher than 0.5, indicating that at least 50% of the variance in each block of indicators can be attributed to the pertinent latent variables [118,128]. Moreover, the values of the Cronbach α are in the range of .60 or higher, thus demonstrating the internal reliability consistency of each construct [119]. Finally, the composite reliability values for each construct are higher than .70, which is the recommended cutoff to validate the internal reliability consistency of each construct relative to all other constructs in the model [128].

Finally, as part of the measurement model, we assessed the possibility of the common method bias using three different procedures.

First, the Harman post hoc one-factor test [121] was conducted. Principal component factor analysis (unrotated solution) revealed 6 factors extracted, with the first factor accounting for 27.3% of the variance. Common method bias was not deemed to be a serious problem with the data because multiple factors emerged, and no single factor accounted for a majority of the variance [121,129].

We subsequently applied the procedure specified by [122] and examined the latent variable correlation matrix from our PLS analysis. Usually, interconstruct correlations of over 0.90 indicate common method variance. In our data, the positive correlations ranged from 0.02 to 0.63, with no observed correlations exceeding the 0.90 threshold. Furthermore, the existence of several low correlations below 0.10 among some of the model constructs indicated that there was no single factor that influenced all constructs [122].

Finally, we used the PLS-based common method bias test suggested by Liang et al [123]. A method factor measured using indicators from all model constructs was added to the research model, and the variance of each item was then explained by its principal construct and method factor. Our results showed that the average variance explained by the principal constructs was 65.2%, whereas the average variance explained by the method factor was 21.5%. The ratio of substantive variance to method variance was approximately 3:1, suggesting that although there may be some common method variance, it does not account for the majority of the variance explained by the model.

Overall, the assessment of the measurement model was deemed satisfactory in terms of item reliability and discriminant validity, and the model constructs were considered to be internally consistent as a measurement scale.

### Structural Model Assessment

Following the measurement model assessment, the structural model was estimated to provide details of the strengths of the relationships among the latent constructs and the overall predictability of the endogenous latent variables in the model.

**Figure 2:**
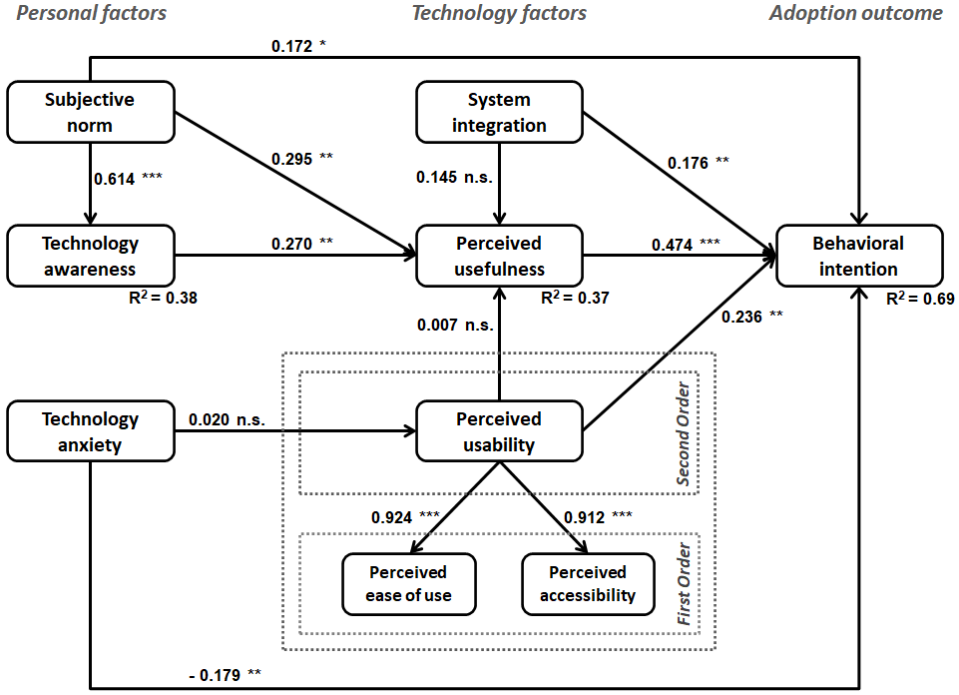
Estimated structural model. *P<.05, **P<.01, *P*<.001; n.s.: not significant at the .05 level.

To estimate the structural model, path coefficients and significance levels were obtained by running PLS with bootstrapping using 1000 resamples. The structural model and *P* values are presented in Figure 2, with path β coefficients depicted along each path. As shown in Figure 2, 8 of the 11 hypotheses were supported with high degrees of confidence, and the model emerged as a good predictor of intention to adopt PHRs, as evidenced by the coefficient of determination (R^2^) value of 0.69 for the ultimate criterion variable. The results are discussed next.

With respect to *personal factors*, a significant relationship was validated between subjective norm and behavioral intention to adopt PHR technologies (H1 supported). In addition, as predicted, subjective norm had a significant positive effect on perceived usefulness and technology awareness (H2 and H3 supported). The path from technology awareness to perceived usefulness was also supported by the model (H4 supported). In terms of the effects of technology anxiety with PHRs, no significant association was found with perceived usability (H5 not supported), but a direct relationship with behavioral intention to use PHR technologies was validated (H6 supported).

The results pertaining to *technology factors* indicate that, contrary to expectations, system integration did not have a direct effect on the perceived usefulness of PHR technologies (H7 not supported). However, system integration was shown to have a direct impact on the user behavioral intention to adopt PHR technologies (H8 supported). As expected, perceived usefulness was shown to be a strong predictor of behavioral intention (H9 supported). With respect to perceived usability, we found an unexpected result of no significant relationship with perceived usefulness (H10 not supported). However, the direct effect of perceived usability on behavioral intention was validated in our model (H11 supported). Further comments on these results are provided in the *Discussion* section.

To determine the efficacy of the model in terms of predictability and goodness of fit (GoF), the coefficients of determination (R^2^) and average communality (AVE) for each construct were evaluated. Together, these measures were used to calculate the global criterion of GoF, as recommended by several researchers [130,131]. Multimedia Appendix 4, Table S4 provide the R^2^ values for all inner model constructs along with their average communalities and the calculated GoF index.

The R^2^ values suggest that the model performed well for the endogenous variables pertaining to technology awareness, perceived usefulness, and behavioral intention. These coefficients of determination (R^2^) explain the proportion of a construct’s variance that can be predicted by antecedent constructs in the model. Most endogenous variables in the model compellingly exceed the minimum threshold of 0.10, indicating the usefulness of that variable in the model [132]. In terms of the ultimate criterion variable in the model, that is, behavioral intention to use PHRs, a significant portion of its variance (around 69%) can be explained by the posited research model.

To calculate the GoF index, the average communality of each construct is calculated as a weighted average of communality (AVE) based on the number of items in each construct taken as its weight [131]. Once calculated, the geometric mean of the average communality and the average R^2^ can be calculated as specified in the GoF formula [131] in Multimedia Appendix 4, Table S4. The suggested baseline values for GoF are 0.1, 0.25, and 0.36 indicating small, medium, or large effect sizes, respectively [133]. As shown in Multimedia Appendix 4, Table S4, the GoF value of our model is 0.480, which exceeds the cutoff value of 0.36 for large effect sizes, allowing us to infer that the model performs well compared with the baseline values of effect sizes. Hence, it can be inferred that the structural model performed well overall.

On the basis of the evaluation of the measurement model validity and reliability, as well as the verification of predictive relevance and GoF of the structural model, we believe that the structural equation model was able to establish a strong basis for relationships posited in the research model hypotheses. Overall, the proposed model acts as an adequate predictor of behavioral intention to use PHRs.

### Content Analysis of Open-ended Responses

As outlined earlier, we asked survey respondents to optionally provide comments about PHRs through textual responses to the question, “Do you have any other comments about the use of personal health records (PHRs)? What factors do you consider to be important in your decision to start using or keep using technologies such as PHRs?”

A total of 63 responses were submitted, and these were analyzed using simple content analysis techniques at the manifest level. In coding and classifying the qualitative data, we searched for themes or concepts related to the adoption of PHRs. An emergent coding technique was used whereby two researchers independently reviewed the responses and created a list of themes and codes. The list was consolidated after mutual consultation. Table 3 summarizes the comments that were classified using this procedure. In the table, we have only shown the three themes that are relevant to our research study—(1) consumer interest in PHR technology as a whole, (2) user interest in specific PHR features (grouped into categories), and (3) user concerns and potential barriers to adoption. It should be noted that each respondent could have contributed to multiple categories through their responses. Therefore, the frequency counts should be interpreted with caution.

**Table 3:** Content analysis summary for open-ended survey responses (n=63).

Themes and comments			Frequency, n (%)
**General consumer interest in PHRs^a^**			40 (64)
	Support the idea of PHRs looking forward to their wider availability	16 (25)	
	PHRs are useful as they provide control or options to patients and their families	12 (19)	
	PHRs useful for chronic illness patients	14 (22)	
	Willing to pay or subscribe for PHR technologies	4 (6)	
**Interest in different PHR features (grouped into categories)**			16 (25)
	Medical information patient and provider records	8 (13)	
	Contact and communication with physician or provider	6 (10)	
	Decision support tools	4 (6)	
	Shared access and social networks	3 (5)	
**Concerns and barriers to adoption**			18 (29)
	Prefer data integration; unwilling to do manual data entry	12 (19)	
	Security and privacy concerns	11 (18)	
	Should be available through mobile apps	6 (10)	

^a^PHR: personal health record**.**

On the whole, many respondents commented on the usefulness of PHR technologies as a whole and indicated their support and anticipation in adopting these technologies. Features related to the maintenance of medical information and online communication with physicians emerged as the most commonly cited PHR functions of interest. Interoperability, security, and privacy issues were frequently mentioned as key factors in the PHR adoption decision. Finally, some respondents stated their interest in using PHR technologies through mobile apps, hence alluding to the notion of accessibility as an important consideration for them.

## Discussion

### Overview

The results outlined in the previous section corroborate the general premise that a combination of personal and technological factors plays a role in determining the adoption of PHR technologies. In exploring these factors, our study has attempted to integrate constructs related to social influence beliefs (subjective norm), individual affective states (awareness and anxiety), and cognitive instrumental perceptions (system integration, perceived usability, and perceived usefulness) that potentially impact adoption behavior (behavioral intention) toward PHR technologies. This section provides an interpretation of the results and discusses the implications for research and practice.

### Personal Factors

Our results indicate that a person’s judgment of subjective norms pertaining to the use of PHR systems plays an important role in the adoption of these technologies through multiple cognitive and affective processes. Its direct impact on behavioral intention suggests that social influence plays a role in people’s decision to adopt PHR technologies. The relatively weak association between subjective norms and behavioral intention can also be explained with reference to past research that shows that subjective norm does not factor prominently as a direct antecedent of behavioral intention in situations where the use of technology is voluntary [68]. This is certainly the case for most users of PHRs. In comparison, there is a stronger association between subjective norm and perceived usefulness, which suggests that internalization of social influence plays a far more important role in the context of PHR adoption. Internalization refers to the process by which a user incorporates the beliefs of an important referent into one’s own belief structure [134]. What this means in the case of PHRs is that the consumers are more likely to develop their own perceptions about the usefulness of these technologies through information they receive from other important people, and this in turn can foster their intention to use PHR systems. Our model also shows that social influence through favorable subjective norms can improve an individual’s awareness of PHR technologies. Overall, subjective norms seem to be an important factor in cognitive and affective mechanisms that allow an individual to make sense of the purpose and benefits of PHR systems.

The positive impact of technology awareness on perceived usefulness also alludes to a process of internalization whereby consumers’ familiarity with the various use cases of PHR technologies allows them to develop beliefs about the technology’s overall usefulness to them. Because the use of PHR systems is voluntary, it is reasonable to assume that consumers would take time to discover and understand the technology before deciding to adopt it. Once again, the relationship between subjective norms and technology awareness implies that observations and interactions with other people play an important role in this process.

Our results also support the critical role of technology anxiety as a determinant of PHR system adoption. Although no significant relationship emerged between technology anxiety and perceived usability, the construct exhibited a significant direct impact on behavioral intention to adopt PHR technologies. With respect to the former, although recent IS studies have shown anxiety to be an important antecedent of perceived ease of use [91,92], our study did not support this relationship. This finding can be attributed to a difference in the type of technology being investigated, as previous studies have generally focused on mandatory use or hedonic technologies. In the case of PHR applications, the technologies are expressly voluntary and instrumental for most consumers. It should also be noted that in adopting the current conceptualization of technology anxiety from the extant IS literature, we might have overlooked the multidimensional nature of anxiety as a psychological construct. Aligned with the IS literature, our construct conceptualization is reflective of anticipatory anxiety (apprehension preceding the use of PHR systems) rather than situational anxiety (distress during the use of PHR systems). The latter may indeed exhibit a relationship with perceived usability. Therefore, we recommend that the multidimensional nature of technology anxiety and its role in the adoption of PHR systems be investigated in future research.

### Technology Factors

The construct of system integration was theorized in our research to measure the importance that users confer on interoperability (among PHRs and other back-end EHR or EMR systems) in their decisions to adopt PHR technologies. Our results demonstrate a positive association between consumer beliefs about PHR interoperability and the intention to adopt these technologies. However, the lack of support for the relationship between system integration features and perceptions of the usefulness of PHR technologies is counterintuitive. In the context of PHRs, it can be expected that better functionality of these systems in terms of connection and interoperability with other back-end systems would translate into better perceptions of the system’s usefulness. This posture is supported by current research on PHR systems that consider a lack of integration between patient-facing systems and back-end eHealth systems as a barrier to adoption for both consumers and health care professionals [21,135].

These differential effects of system integration beliefs can be explained in the context of user expectations. It may be the case that given today’s vast user experience with web-based tools and the pervasive deployment of web services linking different web-based systems, users simply expect PHR systems to be interoperable at the outset. Their common perception about PHRs would align with tethered and interconnected system models of PHRs, and it is these types of technologies that users are interested in adopting. Consumers may factor in these aspects of interoperability only during the initial stages of adoption, and these features are not internalized over time into higher-order cognitive states that represent perceptions of the usefulness of the system. As such, in our research model, the system integration construct is conceptualized in the form of initial expectations pertaining to PHR technologies, and it does not capture or measure aspects of assimilation of these technologies. Therefore, we suggest that future studies use a different approach to model the relationship between system integration and perceived usefulness. One possibility may be to draw upon the experience-disconfirmation theory, which has its roots in consumer behavior research [136], and posits that beliefs and behaviors result from the congruence between expectations and experiences [137].

Unlike many studies investigating technology adoption, our study did not find a significant relationship between perceived usability and perceived usefulness. Although this finding may be at odds with the general IS literature, the findings are not completely surprising in the specific context of PHR system adoption. Previous studies on PHR technology adoption have also shown varied results regarding the effects of perceived ease of use. Some studies confirm construct relationships as defined in the original TAM [107], whereas others contradict them [138]. We offer a possible explanation for this lack of a significant relationship by noting that PHR systems are characterized by their voluntary and instrumental use by potential end users, which requires an extended commitment on the part of end users to keep the system up-to-date and relevant and useful over time. Such systems have recently been the subject of IS research under the category of high maintenance ISs [139]. Initial research on high maintenance ISs contends that usability or ease of use may not be a prominent determinant of usefulness and behavioral intention, as its effect is usually superseded by the effect of other variables such as perceived maintenance effort [139]. In the case of PHR technologies, we expect a greater role for a construct, such as perceived maintenance effort, and future studies should incorporate this variable in their models.

In terms of direct effects on behavioral intention to adopt PHR systems, our results are consistent with the extant research literature. The role of perceived usability and perceived usefulness as antecedents of behavioral intention to adopt PHR systems was validated. Furthermore, having demonstrated internal reliability and construct validity, our integrated conceptualization of perceived usability as a combination of perceived ease and accessibility shows promise in the context of studying PHR technologies. Conceptualization lends support to many researchers’ viewpoints on the synergistic relationship between usability and accessibility [2,65,99].

Responses to technographic questions and the open-ended questions in our survey also reveal consumer preferences for specific PHR features and functions. Our findings contribute to answering the call by other researchers, such as [57], who had asked future researchers to verify their own findings that consumers prefer health care process management support functions, such as communication and contact tools, more than other types of PHR tools. Our research verifies that these tools are among the most preferred tools, along with the category of tools that facilitate the maintenance of patient and provider records. Our findings show patient and provider records in PHRs to be the most preferred category of features, followed by communication and contact features. However, at least until the time when PHR adoption reaches its tipping point, we agree with other research studies that tools related to messaging, appointments, and prescription refills will remain the top-priority features for potential adopters of PHR technologies [140,141].

### Implications for Research

Future studies should further investigate the role of norm internalization and technology assimilation as individual psychological processes affecting behavior toward PHR technologies. We suggest that the relationships among sociotechnical constructs reflect a gradual process in the development of beliefs about PHR technologies and their consequent adoption. For example, in this study, our results suggest that subjective norm and technology awareness are key constructs that affect the consolidation of individual and social values into higher-order cognitive beliefs about the purpose of the benefits of PHR technologies, that is, the internalization process. In the same vein, technology attributes, such as system integration and usability, feature more prominently in the affective and cognitive processes pertaining to technology assimilation. As a possible avenue for future investigations, we believe that incorporating mediating constructs from experience-disconfirmation theory could provide potentially valuable insights into PHR adoption research.

Future research should also seek to explore and validate the potentially multidimensional nature of some of the personal constructs posited in our theoretical model. Specifically, technology anxiety should be studied in terms of anticipatory and situational anxiety. We believe that both of these dimensions play an important role during the different stages of adoption of PHR technologies. Similarly, on the technology side, system integration should be operationalized through specific attributes of integration, such as single window patient information access, system-to-system health data sharing, and information communication capabilities, such as patient-physician exchanges. Doing so would also have the added benefit of deconstructing the specific needs and preferences of consumers in terms of their expectations of integration features and functions between PHR technologies and other HISs.

Our research also provides opportunities to improve health technology assessments. The conceptualization of the two new technology factors of system integration and perceived usability offered in our study may help enhance future systematic evaluations of health care technology. As highlighted earlier, our research shows that functionality, ease of use, and accessibility all play an important role in the adoption of PHR technologies.

### Implications for Practice

In terms of practical implications, our research offers recommendations for PHR technology developers and designers, solution vendors, clinicians, and health policy makers.

Our study highlights the importance of system integration as a significant element affecting the initial decision to adopt PHRs. Technology developers should aim to incorporate interoperability as much as possible. Given the various challenges that exist in achieving seamless point-to-point integration across various types of HISs, developers and vendors should consider the use of health information exchanges as a viable alternative. Industry research suggests that health information exchanges may provide a practical solution to ensuring consumer access to comprehensive longitudinal health records from across the health care delivery chain [80,94].

PHR technology designers should also strive to incorporate accessibility as an element of overall PHR usability. In addition to being easy-to-learn and efficient-to-use, PHR tools should be available through a variety of channels, such as desktop, web, and mobile. Furthermore, PHR systems should facilitate help options and learning pathways to assist end user interactions with the technology features of PHR systems and to support a gradual learning curve. Technology should be developed in such a way as to mitigate anticipatory and situational anxiety with PHR technologies, and it should help end users feel in control of the system. A delineation of basic versus advanced features, context-sensitive suggestions for tasks and actions, and readily available technical support may help alleviate user anxiety and support the adoption of PHR systems [100].

Technology vendors can also help improve the uptake of their PHR systems by influencing personal affective and cognitive beliefs that influence behavior toward PHR technologies. For example, technology awareness can be improved and technology anxiety can be reduced by incorporating additional aspects of trialability and observability in PHR offerings. The availability of free trial versions or free subscriptions, interactive demonstration vignettes and how-to-use videos, access to a community of end users, and spotlights on positive consumer stories can provide useful mechanisms to help alleviate challenges pertaining to technology anxiety and awareness.

Health care providers and practitioners can help improve the uptake of PHR technologies by integrating these tools into clinical encounters and by engaging patients with the technology along various touchpoints in care delivery. The long-term benefits expected from the effective use of these technologies could potentially outweigh any increase in the short-term workload experienced by practitioners in helping promote these technologies to their patients.

From a policy perspective, relevant government agencies can prioritize training and development initiatives for people to become more proficient with the use of PHR systems. The target audience for such programs could include both consumers and health care professionals. The latter factor into the technology adoption process as key influencers as their engagement with patients and their endorsement of relevant PHR applications can accelerate the uptake of these technologies. Government-sponsored technology demonstrations can be administered at community centers or libraries to help improve literacy about PHR technologies, thereby improving consumer awareness, reducing anticipatory anxiety, and leading to greater adoption of these systems. Finally, at the infrastructure level, governments can accelerate the development of interoperability and health data interchange standards that would help make these systems more attractive to consumers and enable faster mainstream adoption.

### Applicability Checks

To further confirm the relevance of our research to the health care sector, we performed applicability checks with several health care professionals, including two physicians, one hospital administrator, one system developer, and one health policy analyst. Applicability checks have been recommended as a useful method for researchers to improve communication between research and practice [142] and substantiate the practical relevance of research [143]. In conducting applicability checks for this research, we sought feedback on our research findings from health care professionals and asked them to comment on the importance of the issues identified in our research. A summary of key comments from the applicability check participants is included in Table 4. Overall, the participants indicated that research studies such as ours could potentially help improve the effective uptake of PHRs and produce efficiencies in the health care system. Furthermore, they commented on the potential of our research to help overcome PHR adoption barriers through actionable guidelines for the health care sector.

**Table 4:** Applicability checks and comments from health care professionals.

Health care professional	Perspective	Key comments
General practitioner (family medicine)	PHR^a^ adoption for improved clinical health outcomes	“PHRs can be great tools to allow patients to become more informed about their conditions and treatments.” “I believe that we can help patients get familiar with the benefits of PHRs and also help them get over their initial hesitation in using these tools.”
Primary care physician (pediatrics)	PHR adoption for improved clinical health outcomes	“I think PHR tools can be great for parents to keep track of their children’s medical history. The information can later be handed over to children once they are able to manage it themselves.” “Once the technical hurdles are resolved, I think clinicians can play an important role in encouraging people to use these technologies. However, we [physicians] have to start using them too and lead by example.”
Hospital administrator (director of operations)	PHR adoption for ensuring continuity of care	“We currently provide access to patients to a limited part of their medical records. Having an integrated medical record across healthcare organizations can be very useful for timely interventions.” “As pointed out in this research, there are many technical obstacles to providing an integrated medical record and this probably hurts overall adoption.”
Systems developer (EHR^b^ systems; mobile health apps)	Functionality and usability requirements for PHR adoption	“Providing access to patient information across organizations is a challenge. Various industry standards are attempting to resolve this issue. Once the problems are resolved, we can expect more user interest in these technologies.” “I agree that usability is more than just thinking about user-friendliness. Users today expect anytime anywhere access to information. This applies to PHRs as well.”
Health policy analyst (digital health strategies)	eHealth initiatives and PHR adoption	“There is a lot of work going on at the national and provincial levels to create the right conditions to support potential applications of PHR technologies.” “Suggestions made in this research can be useful in creating more awareness at the user level. Ultimately, we would like to see PHRs as a technology for all citizens.”

^a^PHR: personal health record.

^b^EHR: electronic health record.

### Study Limitations

As an exploratory study, our research has inherent limitations in terms of the posited research model. This includes hypotheses that did not emerge as significant. Another limitation of our study pertains to the use of convenience and self-selection sampling techniques. This may limit the generalizability of the results of this study. Furthermore, most of the respondents comprised a relatively younger age demographic from North America, and the results may not be representative of the general population.

We also note that by virtue of soliciting responses from a current PHR portal site, health information websites, and forums, our data were collected from respondents with some level of previous interest in health self-management. This limits our findings to current internet users with potentially higher health literacy and may not accurately account for the population of users with less exposure to health information or with less access to computing resources. Future research should include potential and actual users of PHR technologies through more diversified sources and utilize recruitment mechanisms to alleviate sampling bias.

### Conclusions

Advancing the use of technologies in all walks of life is also increasing people’s expectations of user-centered health care technologies. Consequently, consumer demand for PHR systems is likely to remain strong in the upcoming years. Recent academic and industry research on PHR systems has affirmed abundant consumer interest in these technologies [4,80,94].

The empirical research findings reported in this paper aim to contribute to the body of knowledge on consumer adoption of PHRs. To this end, we have attempted to explore and analyze possible factors contributing to what has been termed the *PHR paradox* [21], that is, despite their predicted benefits and considerable consumer interest, the adoption of PHRs has generally remained low. Our study also answers the call for researchers to investigate the facilitators and inhibitors of PHR adoption at multiple levels, including personal and technological [2,21,51,66].

By developing and validating a parsimonious research model comprising personal and technological determinants of PHR adoption, we were able to obtain several insights into the social influence and cognitive instrumental processes that impact consumer adoption of PHRs. Our results indicate that subjective norms, technology awareness, and technology anxiety are important factors that predict individual attitudes and beliefs about the usefulness of PHR systems and the ultimate adoption of these technologies. Our study also shows the differential effects of system integration capabilities and perceived usability on perceived usefulness and behavioral intention to adopt PHRs. Our characterization of PHR technologies in terms of their voluntary, instrumental, and high maintenance attributes has allowed us to make sense of some of the seemingly counterintuitive findings about technology antecedents of PHR adoption.

As such, our findings support the viewpoint of other researchers who contend that PHR technologies are complex innovations in which perceived attributes of technology are neither stable features nor sure determinants of adoption [21,95]. We encourage future research to examine the adoption of PHRs in a longitudinal fashion, exploring the role of different sociotechnical factors affecting users’ cognitive and behavioral processes during the stages of internalization, assimilation, and maintenance of PHR systems.

We hope that the takeaways from our study will prove to be constructive in helping align PHR offerings more closely with consumer beliefs and attitudes, as well as their informational needs and functional requirements. This should help alleviate the risk of PHR technology rejection or abandonment.

## Appendix

Multimedia Appendix 1 Literature review summary.

Multimedia Appendix 2 Psychometric scales and measurement indicators.

Multimedia Appendix 3 Summary of responses to technographic questions.

Multimedia Appendix 4 Measurement and structural model assessment.

## Abbreviations

AVE: average variance extracted
CHI: consumer health informatics
EHR: electronic health record
EMR: electronic medical record
GoF: goodness of fit
HIS: health information system
IS: information system
PHR: personal health record
PLS: partial least squares
SEM: structural equation modeling
TAM: technology acceptance model
