# Supplementary material for: Sociotechnical Drivers and Barriers in the Consumer Adoption of Personal Health Records: Empirical Investigation
Source: JMIR Med Inform. 2021 Sep 24;9(9):e30322. doi: 10.2196/30322 (PMC8501412; doi:10.2196/30322)
Supplement: Multimedia Appendix 1 [file medinform_v9i9e30322_app1.docx]

**APPENDIX 1 - Literature Review Summary**

| **Literature Source** | **Type of Research &  Study Context** | **Highlighted  PHR Adoption Factors** | **Key  Take-aways** |
| --- | --- | --- | --- |
| [46] | - Empirical, Qualitative - Tertiary care center PHR for chronically ill patients | Promotion of Sense of Illness Ownership; Patient-Driven Communication; Personalized Support; Mutual Trust | - A patient-centered framework for improving the perceived usefulness of PHRs should concentrate on enhancing the four factors identified in this study. |
| [8] | - Conceptual - Authors’ reflections | Illness status (well, chronic, acute etc.); Awareness; Behavioural Change | - Chronically ill patients and those aware of PHR benefits are more likely to adopt these technologies. - Further empirical research is needed to explore other adoption factors. |
| [50] | - Empirical, Qualitative - General Internet users including patients and health professionals | Comprehensive patient medical information; Communication with Clinicians; Privacy | - Factors listed constitute key drivers for PHR adoption. - Privacy issues are not as much a concern for the chronically ill, and generally less of a concern to patients than to health professionals. |
| [39] | - Empirical, Qualitative - PHR system demonstration to consumers, clinicians and administrators at a community health organization | Awareness; Expectations; Privacy; Autonomy | - Low awareness and high consumer expectations are potential barriers to PHR adoption. - More research is needed in this area. |
| [53] | - Empirical, Mixed Methods - Institutional-neutral PHR system provisioned for low-income elderly residents on a housing facility | Health Literacy; Computer Self-Efficacy; Anxiety | - Factors listed constitute key barriers in PHR adoption. - Additional research is needed to explore role of Anxiety as a major deterrent in PHR adoption. |
| [55] | - Empirical, Quantitative - Tethered PHR for patients at an HIV/AIDS clinic | Ease of Use; Data Accuracy; Privacy | - Factors listed contribute positively to the adoption of PHRs. |
| [26] | - Systematic Literature Review | Various PHR adoption factors from the extant literature were reviewed | - More research on consumer adoption of PHRs is needed, especially on functionality and usability related factors. |
| [24] | - Empirical, Quantitative - Consumers of tethered PHRs from two medical centers | Relative advantage; Ease of Use; Trialability; Privacy and security; Computer use | - Factors listed contribute positively to the adoption of PHRs. |
| [44] | - Empirical, Qualitative - Type-2 diabetes patients using PHR of a managed care facility | Real-time Decision Making Support; Information Exchange with Clinicians; Support Groups | - Features listed are most commonly desired functions in PHRs for diabetes self-management. |
| [52] | - Empirical, Quantitative - Pilot program for a USB-PHR distribution across medical facilities in Taiwan | Perceived Usefulness; Perceived Ease of Use; Subjective Norm; Security & Privacy; Computer Self-Efficacy; Behavioral Intention | - Perceived Usefulness and Subjective Norm were found to be significant determinants of PHR adoption intention. - Future research should explore effects of usability and interoperability perceptions on adoption. |
| [47] | - Empirical, Qualitative - USB and Web-based PHR systems for HIV/AIDS patients at two primary care facilities | Efficiency; Provider Communication; Computer Access; Computer Literacy | - Efficiency and Provider Communication benefits act as drivers for PHR adoption, while computer access and computer literacy are potential barriers. |
| [57] | - Empirical, Quantitative - Live tethered PHR for patients of a medical facility | Satisfaction with Healthcare Provider; Patient Activation; Perceived value of PHR functionality; Perceived empowerment potential of PHR; Communication tactics | - Interaction between various posited factors was shown to impact PHR adoption intention. - Future research encouraged on role of communication tactics, and to verify patient preferences for healthcare process management support functions. |
| [48] | - Empirical, Qualitative - Web-based PHR systems for HIV/AIDS patients | Computer Access; Computer Self-Efficacy; Privacy | - Factors listed constitute key barriers in the adoption of PHRs. |
| [22] | - Empirical, Quantitative - Internet panel of chronically ill patients | Internet reliance; Computer Self-Efficacy; Personal IT innovativeness; Anxiety; Access to Data sources; Satisfaction with medical care; Information-seeking; Perceived usefulness; Security, privacy and trust; Behavioral intention | - Most hypotheses related to antecedents of usefulness and behavioral intention were validated, with a few inconsistent findings between the two studies. - Relationship between computer self-efficacy and perceived usefulness was not supported in [21]. - Neither study performed hypothesis validation between self-efficacy and anxiety, or anxiety and usability. |
| [51] | - Empirical, Quantitative - Internet panel of chronically ill patients and healthy consumers | Computer Anxiety; Information seeking; Personal IT innovativeness; Security, privacy and trust; Perceived usefulness; Behavioral intention |  |
| [25] | - Systematic Literature Review | Various PHR adoption factors from the extant literature were reviewed | - More research on consumer adoption perceptions is needed. - Awareness and usability related research is especially lacking in the current literature. |
| [54] | - Systematic Literature Review | Encouragement by care provider, access to and control over health data, increased communication with care provider | - Three facilitators and barriers to PHR use. - Future research on effective PHR education strategies, and a framework to identify ideal outcome measures from PHR use. |
| [56] | - Empirical, Qualitative - Definition of ePHR - Stakeholder perceptions of barriers and facilitators to ePHR adoption | Knowledge, system design, user capabilities/attitudes, environmental factors, legal/ethical issues | - No consensual definition - Future research required to establish definition of ePHR, clarification of data ownership and interoperability. |
| [49] | - Empirical, Quantitative - Awareness of patient portal and interest in portal functionalities | Majority of patients interested to view personal health data; half of participants interested in secure messaging | - Unawareness of portal main reason for lack of use - More research required to assess impact of portal use on compliance to treatment. |
| [45] | - Empirical, Quantitative - Theoretical model to explain middle-age and elderly use of PHR | Perceived severity, benefits, self-efficacy and calls to action effect intention to use PHR | - First study to use existing health belief theory to investigate middle-age and elderly PHR use. - Study should be replicated with larger sample size and other population groups |
| [41] | - Empirical, Quantitative - Rate of PHR adoption over time | Blacks and other races equally likely to use PHRs, along with those with higher education, higher internet usage, and those using prescription medication | - Hispanics and patients with Medicaid less likely to use PHRs - More outreach required to enable disadvantaged groups |
