# Supplementary material for: Sociotechnical Drivers and Barriers in the Consumer Adoption of Personal Health Records: Empirical Investigation
Source: JMIR Med Inform. 2021 Sep 24;9(9):e30322. doi: 10.2196/30322 (PMC8501412; doi:10.2196/30322)
Supplement: Multimedia Appendix 2 [file medinform_v9i9e30322_app2.docx]

**APPENDIX 2 – Psychometric Scales & Measurement Indicators**

| **Theoretical Construct** | **Measurement Indicators** | **Source** |
| --- | --- | --- |
|  |  |  |
| **Subjective Norm** | - Most people who are important to me think I should use PHR systems. - The people I listen to [could influence / have influenced] me to [start using / use] PHR systems. - Close friends and family members think it is a good idea for me to use PHR systems. | Adapted from [68] |
| **Technology Awareness** | - I am aware of the benefits of personal health records as a self-health management tool. - I know what a PHR is and how it works. - I have enough information in order to decide to use PHR for my health management. | Adapted from [73] |
| **Technology Anxiety** | - I [would / ] hesitate to use a PHR system due to fear of making mistakes I cannot correct. - I [would / ] feel nervous in using PHR technology applications for accomplishing tasks. - I have low confidence in my skills to use PHR technologies. | Adapted from [71] |
| **System Integration** | - [I expect / ] PHR systems [to / ] provide access to my entire health records in one place. - PHR applications [would / are] usually [be / ] integrated with other electronic health records. - My PHR [should be / is] able to send and receive information from other systems that keep my health data. | New scale  in this study |
| **Perceived Usability** | - I believe [a / the] PHR system [should be / is] easy to use. - I think it [should be / is] easy to interact with PHR systems. - PHR systems [should be / are] easy to learn. - Information from PHR systems [should be / is] accessible anywhere anytime. - PHR systems [should / ] present information in a clear fashion. - PHR systems [should be / are] available when I need them. | Ease of Use Scale (first 3 items) adapted from;[68]  Accessibility Scale (last 3 items) new in this study |
| **Perceived Usefulness** | - I believe that PHR systems are a useful technology to manage health conditions. - Using PHRs has a lot of advantages for health management. - PHR systems are capable of providing benefits in individual health management. | Adapted from [68] |
| **Behavioral Intention** | - [If a PHR is made available to me, I intend to use it / I plan to continue using the PHR system]. - I would [like to / ] use a PHR system for keeping my health records up to date. - I would [consider using / use] a PHR system to manage my health information in the future. | Adapted from [68] |
