# Supplementary figures and images for: Sociotechnical Drivers and Barriers in the Consumer Adoption of Personal Health Records: Empirical Investigation
Source: JMIR Med Inform. 2021 Sep 24;9(9):e30322. doi: 10.2196/30322 (PMC8501412; doi:10.2196/30322)

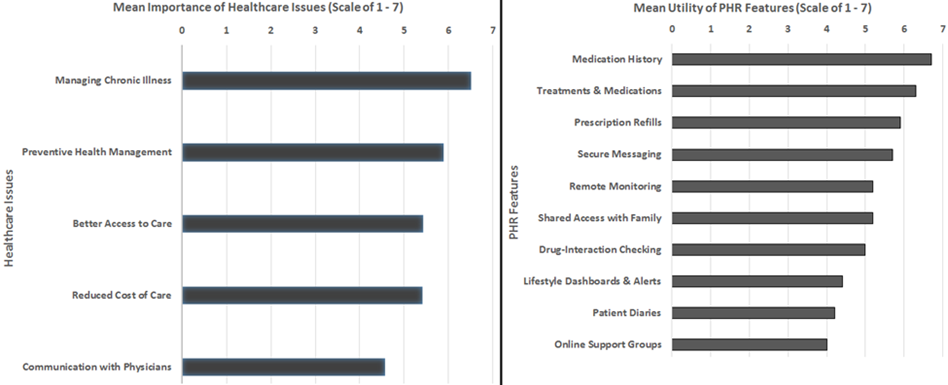

Supplement: Multimedia Appendix 3 [file medinform_v9i9e30322_app3.png]
