# Supplementary material for: Sociotechnical Drivers and Barriers in the Consumer Adoption of Personal Health Records: Empirical Investigation
Source: JMIR Med Inform. 2021 Sep 24;9(9):e30322. doi: 10.2196/30322 (PMC8501412; doi:10.2196/30322)
Supplement: Multimedia Appendix 4 [file medinform_v9i9e30322_app4.docx]

**APPENDIX 4 – Measurement & Structural Model Assessment**

**Table S1.** Item Level Discriminant Validity Assessment: Matrix of Loadings & Cross-Loadings in the Outer Measurement Model

| **Measurement Items** | **Model Constructs** | | | | | | | |
| --- | --- | --- | --- | --- | --- | --- | --- | --- |
|  | **SN** | **TAw** | **TAnx** | **SI** | **PU** | **PEoU** | **PA** | **BI** |
|  |  |  |  |  |  |  |  |  |
| S_N_1 | **0.777** | 0.472 | -0.085 | 0.401 | 0.574 | 0.483 | 0.517 | 0.57 |
| S_N_2 | **0.724** | 0.493 | 0.298 | 0.439 | 0.154 | 0.42 | 0.419 | 0.241 |
| S_N_3 | **0.761** | 0.421 | -0.013 | 0.479 | 0.38 | 0.545 | 0.433 | 0.511 |
| T_Aw_1 | 0.507 | **0.867** | 0.077 | 0.468 | 0.525 | 0.548 | 0.538 | 0.367 |
| T_Aw_2 | 0.536 | **0.847** | 0.133 | 0.349 | 0.464 | 0.534 | 0.495 | 0.384 |
| T_Aw_3 | 0.457 | **0.728** | 0.163 | 0.405 | 0.247 | 0.466 | 0.511 | 0.284 |
| T_Anx_1 | 0.019 | 0.111 | **0.737** | -0.036 | -0.103 | -0.106 | -0.037 | -0.153 |
| T_Anx_2 | 0.007 | 0.086 | **0.893** | 0.027 | -0.167 | 0.036 | 0.026 | -0.238 |
| T_Anx_3 | 0.109 | 0.188 | **0.694** | 0.205 | -0.05 | 0.076 | 0.13 | -0.099 |
| S_Int_1 | 0.392 | 0.402 | 0.059 | **0.794** | 0.429 | 0.423 | 0.33 | 0.369 |
| S_Int_2 | 0.433 | 0.372 | 0.041 | **0.812** | 0.31 | 0.562 | 0.445 | 0.463 |
| S_Int_3 | 0.536 | 0.397 | 0.041 | **0.784** | 0.328 | 0.545 | 0.512 | 0.534 |
| P_U_1 | 0.38 | 0.302 | -0.263 | 0.314 | **0.824** | 0.386 | 0.285 | 0.615 |
| P_U_2 | 0.55 | 0.55 | -0.021 | 0.428 | **0.861** | 0.443 | 0.37 | 0.623 |
| P_U_3 | 0.407 | 0.425 | -0.122 | 0.367 | **0.794** | 0.407 | 0.295 | 0.603 |
| EoU_1 | 0.566 | 0.548 | 0.065 | 0.579 | 0.438 | **0.812** | 0.595 | 0.465 |
| EoU_2 | 0.514 | 0.509 | 0.001 | 0.559 | 0.402 | **0.908** | 0.641 | 0.55 |
| EoU_3 | 0.601 | 0.519 | -0.053 | 0.544 | 0.453 | **0.861** | 0.638 | 0.623 |
| Acc_1 | 0.563 | 0.572 | 0.059 | 0.354 | 0.359 | 0.566 | **0.801** | 0.393 |
| Acc_2 | 0.346 | 0.416 | 0.079 | 0.456 | 0.243 | 0.513 | **0.765** | 0.372 |
| Acc_3 | 0.549 | 0.553 | -0.039 | 0.496 | 0.314 | 0.613 | **0.815** | 0.496 |
| B_I_1 | 0.519 | 0.247 | -0.311 | 0.384 | 0.544 | 0.37 | 0.289 | **0.755** |
| B_I_2 | 0.457 | 0.448 | -0.048 | 0.521 | 0.591 | 0.595 | 0.492 | **0.796** |
| B_I_3 | 0.502 | 0.313 | -0.183 | 0.472 | 0.461 | 0.527 | 0.485 | **0.810** |
| **Model Constructs:** SN: Subjective Norm ; TAw: Technology Awareness ; TAnx: Technology Anxiety ; SI: System Integration PU: Perceived Usefulness ; PEoU: Perceived Ease of Use ; PA: Perceived Accessibility ; BI: Behavioral Intention | | | | | | | | |

**Table S2.** Construct Level Discriminant Validity Assessment: AVEs & Inter-Construct Correlations in the Measurement Model

| **Constructs** | **BI** | **PEoU** | **PA** | **PU** | **SN** | **SI** | **TAnx** | **TAw** |
| --- | --- | --- | --- | --- | --- | --- | --- | --- |
|  |  |  |  |  |  |  |  |  |
| **BI** | **0.788** |  |  |  |  |  |  |  |
| **PEoU** | 0.636 | **0.861** |  |  |  |  |  |  |
| **PA** | 0.533 | 0.726 | **0.794** |  |  |  |  |  |
| **PU** | 0.742 | 0.500 | 0.386 | **0.826** |  |  |  |  |
| **SN** | 0.627 | 0.650 | 0.618 | 0.546 | **0.746** |  |  |  |
| **SI** | 0.583 | 0.651 | 0.550 | 0.451 | 0.581 | **0.787** |  |  |
| **TAnx** | -0.231 | 0.004 | 0.038 | -0.154 | 0.041 | 0.059 | **0.765** |  |
| **TAw** | 0.428 | 0.635 | 0.651 | 0.524 | 0.614 | 0.497 | 0.146 | **0.814** |
| **Model Constructs:** BI: Behavioral Intention ; PEoU: Perceived Ease of Use ; PA: Perceived Accessibility ; PU: Perceived Usefulness ; SN: Subjective Norm ; SI: System Integration ; TAnx: Technology Anxiety ; TAw: Technology Awareness | | | | | | | | |

**Table S3.** Convergent Validity Assessment of the Measurement Model

| **Latent Variables** | **Convergent Validity Indicators** | | |
| --- | --- | --- | --- |
|  | **Average Variance Extracted (AVE)** | **Composite Reliability** | **Cronbach’s Alpha** |
| Behavioral Intention | 0.614 | 0.827 | 0.694 |
| Perceived Ease of Use | 0.741 | 0.895 | 0.824 |
| Perceived Accessibility | 0.639 | 0.836 | 0.707 |
| Perceived Usability | 0.621 | 0.898 | 0.864 |
| Perceived Usefulness | 0.683 | 0.866 | 0.768 |
| Subjective Norm | 0.588 | 0.794 | 0.617 |
| System Integration | 0.622 | 0.866 | 0.728 |
| Technology Anxiety | 0.592 | 0.803 | 0.659 |
| Technology Awareness | 0.662 | 0.854 | 0.746 |

**Table S4.** Predictability of Constructs & Model's Global Goodness-of-Fit Index

| **Endogenous Model  Constructs** | **Communality**  **(AVE)** | **Variance Explained**  **(R^2^)** | **Number of**  **Indicators** |
| --- | --- | --- | --- |
|  |  |  |  |
| Behavioral Intention | 0.620 | 0.689 | 3 |
| Perceived Usability | 0.591 | 0.000 | 6 |
| Perceived Usefulness | 0.683 | 0.372 | 3 |
| Technology Awareness | 0.662 | 0.378 | 3 |
| Average R-Square $\bar{R^{2}}$ |  | 0.360 |  |
| Average Communality (Weighted Average) $\bar{AVE}$ | 0.629 |  |  |
| Goodness of Fit (GoF) Index $GoF=\sqrt{\bar{AVE}*\bar{R^{2}}}$ | | 0.480 | |
